# Ocular Angiogenesis

**DOI:** 10.1155/2015/892043

**Published:** 2015-09-28

**Authors:** Juliana L. Dreyfuss, Ricardo J. Giordano, Caio V. Regatieri

**Affiliations:** ^1^Department of Health Informatics, Federal University of São Paulo, Paulista School of Medicine, 04023-062 São Paulo, SP, Brazil; ^2^Department of Biochemistry, Federal University of São Paulo, Paulista School of Medicine, 04039-032 São Paulo, SP, Brazil; ^3^Department of Biochemistry, University of São Paulo, 05508-000 São Paulo, SP, Brazil; ^4^Department of Ophthalmology, Federal University of São Paulo, Paulista School of Medicine, 04023-062 São Paulo, SP, Brazil; ^5^Department of Ophthalmology, Tufts University School of Medicine, Boston, MA 02111, USA

This special issue shows the continuing efforts to understand the molecular biology and the development of new treatments and diagnostic tools for ocular angiogenesis. In this issue we bring novel research and discuss challenges in developing therapeutics for ocular neovascular diseases, angiogenesis and its role in ocular diseases, and the mechanisms leading to progressive vessel dysfunction and blindness.

This issue shows papers describing clinical and experimental studies of ocular angiogenesis (OA), showing advances in molecular biology and new insights into retinal, corneal, and choroidal neovascularization and imaging techniques, besides current concepts in the treatment of ocular angiogenesis. This special issue promotes new ideas, inspires discussion of the concepts presented in the issue, increases the understanding of mechanisms that control the dynamic process of angiogenesis in the eye, and brings together the available information of new types of treatments.

Angiogenesis has fundamental importance in disease and health. It is the formation of new blood vessels from preexisting vasculature. Angiogenesis is a complex process constituting multiple steps. Extracellular matrix degrading enzymes secreted by activated endothelial cells degrade the basement membrane, allowing the migration and proliferation of these cells, resulting in the formation of solid endothelial cell capillary tubes. Pathologic angiogenesis in the eye can lead to severe visual impairment. The ocular angiogenesis can occur in retina, choroid, and cornea. The ocular angiogenesis is related to a broad spectrum of disorders such as wet age-related macular degeneration (AMD), diabetic retinopathy, retinal artery or vein occlusion, retinopathy of prematurity (ROP), neovascular glaucoma, and corneal neovascularization secondary to infectious or inflammatory processes.

Ocular neovascularization is an intricate process controlled by myriad angiogenic agents such as growth factors, cytokines, and extracellular matrix components. Angiogenesis is regulated by a balance between endogenous proangiogenic and antiangiogenic factors. And the diseases where ocular angiogenesis occurs require disruption of such balance; thus, the angiogenic switch must be turned “on” for neovascularization progression.

The proangiogenic growth factors implicated in pathologic vessel formation in ocular diseases are fibroblast growth factor (FGF), platelet-derived endothelial growth factor (PDGF), and vascular endothelial growth factor (VEGF), among others. The antiangiogenic factors are pigment epithelium-derived factor (PEDF), endostatin, and thrombospondin, among others. Identification of these angiogenesis regulators has enabled the development of novel therapeutic approaches for many ocular disorders.

Recent clinical studies regarding the intravitreal injection of monoclonal antibodies anti-VEGF (ranibizumab and bevacizumab) have shown excellent results in the treatment of degenerative and vascular chorioretinal diseases. Diagnostic imaging tools have played an increasingly important role in eye care in recent years.

Advances in fundus imaging make assessment of peripheral neovascularization in many chorioretinal diseases possible, such as diabetic retinopathy and retinal vein occlusion. The technology has unveiled new insights into the role of peripheral pathology in retinal vascular, degenerative, and inflammatory diseases.

Additionally, new techniques in optical coherence tomography have improved the axial image resolution and image acquisition and the ability to allow the detection of individual retinal layers and lesion components. With spectral domain it is possible to identify retinal new vessels in wet age-related macular degeneration using OCT angiography. The next step will be the swept source OCT development, which will make the blood flow measurements possible. The study by W. Chen et al. investigates the aqueous levels of pigment epithelium-derived factor (PEDF) and macular choroidal thickness in individuals with high myopia. The authors noticed a significant correlation between aqueous PEDF levels and macular choroidal thickness in patients without angiogenesis but no association with the group of patients with pathological angiogenesis. It would be interesting to expand these association studies to include vascular endothelial growth factor and assess whether the balance between VEGF and PEDF levels could predict disease progression and resistance to anti-VEGF therapy. Another study by S. Rusnak et al. shows that the concentrations of IL-6, TGF-*β*1, and VEGF correlate with the severity of proliferative diabetic retinopathy and were particularly high in patients with refractive neovascular glaucoma. IL-6 is a multifunctional cytokine with pro- and anti-inflammatory properties and TGF-*β*1 is an interesting therapeutic target for ocular angiogenesis. Their results reinforce these findings and also suggest that, in patients with neovascular glaucoma refractory to treatment, IL-6 and TGF-*β*1 may have a potential use in patient stratification and in determining personalized medical needs.

The mechanisms and new experimental treatments for corneal neovascularization are investigated and presented in this issue. K. Tomoyose et al. show that the loss of TRP vanilloid subtype 1 (TRPV1), the capsaicin receptor, did not affect VEGF-dependent neovascularization in cell culture. On the other hand, lack of TRPV1 inhibited neovascularization in mouse corneal stroma following cauterization. The study performed by J. Yoshida et al. shows the inhibition of corneal neovascularization by subconjunctival injection of Fc-endostatin in rabbit corneas. The endostatin is an angiogenesis inhibitor, a fragment of collagen XVIII, and the Fc-endostatin was developed by fusing endostatin to the Fc region of an IgG molecule. The subconjunctival injection of this antiangiogenic molecule showed to be efficient and safe in rabbit model.

The role of stromal cells in angiogenesis is also addressed in this special issue. The role of molecules in pericytes and anti-VEGF therapy in fibroblasts is shown in this issue. Pericytes are contractile cells that interact with endothelial cells stabilizing the newly formed vessel. These cells modulate vascular permeability and blood flow and can regulate angiogenesis modulating endothelial proliferation, differentiation, and migration. The study by L. Chen et al. shows the protective effect of apelin (an endogenous ligand of G protein-coupled receptor APJ) from apoptosis due to chemical hypoxia induced in rat retinal pericytes. The study performed by L. Lytvynchuk et al. shows the antiproliferative, apoptotic, and autophagic activity of anti-VEGFs on fibroblasts cultures. These effects upon fibroblasts may explain the cellular response and the etiology of choroidal neovascularization involution after treatment with anti-VEGFs.

This special issue additionally presents very interesting clinical studies. R. Mastropasqua et al. reveal that optical coherence tomography-angiography is a noninvasive dyeless method to image the retinal microcirculation; the images provided distinct vascular patterns for different diseases. It provides detailed images of retinal vascular plexuses and quantitative data of pathologic structures. P. Calvo et al. analyzed the visual outcome in 51 patients with wet age-related macular degeneration depending on the number of ranibizumab injections after 3 years of follow-up. The best outcomes were found in stable wet AMD patients that received ≥ 7 ranibizumab intravitreal injections in 3 years. Following the same treatment modality for wet AMD, anti-VEGF therapy using ranibizumab, A. García-Layana et al. compile data showing the comparison of different regimens of intravitreal injections and their outcomes. Finally, but not less interestingly, S. D. Nicoara et al. show the outcomes of bevacizumab treatment of retinopathy of prematurity. They recorded no complications subsequent to the intravitreal injections of bevacizumab with no late retinal detachment. In addition the study shows a very high ROP regression rate after one intravitreal bevacizumab injection.

We expect that the present volume on ocular angiogenesis may provide useful information to understand the mechanisms and new therapies and diagnostic tools for ocular angiogenesis to the readers.

*Juliana L. Dreyfuss* *Juliana L. Dreyfuss* *Ricardo J. Giordano* *Ricardo J. Giordano* *Caio V. Regatieri* *Caio V. Regatieri*

